# Fe-Exchanged Natural Bentonites from Kazakhstan as Multifunctional Solids for Decontamination from Hazardous Chemicals: Structure–Reactivity Relationships Under Mild Conditions

**DOI:** 10.3390/molecules31101771

**Published:** 2026-05-21

**Authors:** Stefano Econdi, Sholpan Nazarkulova, Stefano Marchesi, Chiara Bisio, Mukhambetkali Burkitbayev, Matteo Guidotti

**Affiliations:** 1 CNR–SCITEC, Institute of Chemical Sciences and Technologies “Giulio Natta”, Via C. Golgi 19, 20133 Milan, Italy; stefano.econdi@scitec.cnr.it (S.E.); sholpan.nazarkulova@kaznu.edu.kz (S.N.); chiara.bisio@uniupo.it (C.B.); 2 Faculty of Chemistry and Chemical Technology, Al-Farabi Kazakh National University, 71, Al-Farabi Av., Almaty 050040, Kazakhstan; mukhambetkali.burkitbayev@kaznu.edu.kz; 3 Department of Sciences and Technological Innovation, University of Eastern Piedmont “A. Avogadro”, Viale T. Michel 11, 15121 Alessandria, Italy; stefano.marchesi@uniupo.it

**Keywords:** clays, montmorillonite, ion exchange, iron, heterogeneous oxidation, decontamination, organophosphorus chemicals, organosulfur compounds

## Abstract

Iron-exchanged bentonites derived from a natural montmorillonite-rich clay (Taganskoe deposit, Kazakhstan) were prepared through a simple aqueous ion-exchange route using Fe(II) or Fe(III) inorganic salt precursors, yielding final Fe contents of ca. 5–7 wt.%, while preserving the smectite layered framework. A mild thermal treatment under air was applied to tune iron coordination without triggering major structural collapse. The resulting materials were characterized by ED-XRF, PXRD, FE-SEM/EDX, DLS/ζ-potential and DR UV–Vis–NIR spectroscopy, revealing predominantly exchanged Fe species with a limited fraction of surface iron-oxide clusters, whose contribution increases after activation. Structure–reactivity relationships were probed under mild conditions in liquid-phase ethyl acetate using dimethyl methylphosphonate (DMMP) and 2-chloroethyl ethyl sulfide (2-CEES) as organophosphorus and organosulfur hazardous chemicals and chemical warfare agent simulants, respectively. Fe(III)-bentonite enabled very fast DMMP removal (ca. 93% within 0.5 h) with a remarkable improved performance with respect to Fe(II)-bentonite and the pristine mineral clay. For 2-CEES, the presence of H_2_O_2_ markedly enhanced oxidation on Fe-containing clays, reaching quantitative abatement within 24 h (up to >90%), with strong retention of oxidized sulfur products by the clay matrix. These results highlight Fe-exchanged natural bentonites as robust, cheap and multifunctional adsorption/catalytic solids for decontamination and water-treatment applications.

## 1. Introduction

The persistent risk associated with organophosphorus (OP) and organosulfur (OS) hazardous chemicals spans two traditionally distinct domains: (i) environmental contamination from industrial/agricultural chemicals and their transformation products [1], and (ii) the intentional or accidental release of highly toxic chemical warfare agents (CWAs) or their simulants [2]. This dual focus is dictated by common physico-chemical properties that govern the behavior of both pollutant classes: many OP/OS molecules combine limited biodegradability with good affinity for mineral and polymeric surfaces, and their removal often requires an interplay between adsorption (to concentrate the target at interfaces) and catalysis (to selectively cleave P–O/P–S, C–S or C–Cl bonds without generating persistent secondary waste or even more toxic compounds) [3]. Advanced oxidation processes are among the most versatile approaches to address this challenge, but they face practical difficulties in energy efficiency, oxidant utilization and the control of by-products and catalyst lifetime, especially when translated from laboratory model systems to real water matrices [4,5,6,7]. Iron-mediated Fenton-like chemistry is quite attractive because Fe is earth-abundant and can activate oxidants, like H_2_O_2_ and related peroxides, to generate short-lived but highly reactive oxygen species (ROS). However, the homogeneous Fenton process is limited by a narrow pH window, the need for continuous Fe dosing, and the formation of iron-containing sludge, motivating heterogeneous variants in which Fe is immobilized on solids. Critical aspects for the scale-up of these latter processes include: (i) stabilizing the Fe redox cycle under almost neutral conditions, (ii) mitigating scavenging by natural organic matter and inorganic anions, (iii) evaluating the role of any possible dissolved iron species in relation to the desired heterogeneous degradation pathways and (iv) ensuring catalyst regeneration with minimal secondary emissions [4,5,8,9].

Clay minerals, particularly smectites, can be considered a versatile platform for heterogeneous iron chemistry. Their 2:1 T-O-T (T = tetrahedral, O = octahedral) layered structure provides a relatively high cation-exchange capacity (CEC) and specific surface area, alongside a tunable interlayer environment. Furthermore, the abundance of oxygen functionalities at edge and basal sites offers a rich population of structural defects for coordinating transition metals [10]. Moreover, the worldwide availability, low cost, and proven industrial scalability of natural clays make them ideal candidates for advanced environmental remediation [11]. Therefore, they represent promising platforms for both ‘passive’ adsorption (e.g., heavy metals, dyes, pharmaceuticals) and ‘active’ oxidation catalysis (e.g., toxic compounds such as pesticides or chemical warfare agents, CWAs) [12,13]. This versatility stems from their ability to host redox-active species within the structure and/or in the interlayer space, promoting different degradation pathways, often enhanced by oxidizing molecules such as H_2_O_2_. While ion exchange, surface complexation and interlayer swelling effectively control the uptake of cations and polar organics, adsorption alone merely transfers pollutants from water to a solid phase, falling short of ensuring complete detoxification [14,15].

Consequently, metal-exchanged and metal oxide-modified montmorillonites have emerged as interesting solids, designed to bridge the gap between pollutant adsorption and catalytic degradation [16,17]. Three main strategies dominate the current technological landscape in this field: (i) pillared interlayered clays (PILCs), where polyoxocations (e.g., Ti, Co, Al) act as structural pillars to expand the interlayer gallery, creating stable porous networks that can host catalytically active species; (ii) surface modification via impregnation or deposition of Fe oxy/hydroxides and mixed oxides; (iii) direct ion exchange of hydrated Fe species into the interlayer space, often followed by thermal activation to partially anchor Fe species and modulate hydrolysis/condensation equilibria [18,19,20].

The PILC approach has been extensively used for Fenton-like water treatment, highlighting how increased basal *d*_(001)_ spacing, higher surface area and controlled Fe nuclearity can improve activity while reducing metal leaching. Yet, these benefits are frequently compromised by stringent synthesis requirements and the inherent difficulty of preserving the coordination environment of Fe sites against leaching or aggregation over successive cycles [7,21]. By contrast, direct Fe exchange is experimentally simpler and highly scalable, and the clay interlayer region is used as a confined space for Fe(III)/Fe(II) redox processes. For instance, peroxymonosulfate activation by Fe(III)-saturated montmorillonite has demonstrated rapid atrazine adsorption into the interlayer space followed by degradation via confined Fe redox cycling. This suggests that montmorillonites can modulate the kinetics and selectivity of radical and non-radical pathways by altering local concentrations, dielectric environment and electron-transfer distances [22].

Nevertheless, the use of Fe-intercalated layered materials in heterogeneous oxidation processes has some limitations. Catalytic studies commonly rely on acidic pH conditions (typically ≤3) to maximize Fenton reaction rates, whereas real contaminated waters and media frequently require near-neutral pH environments. On the other hand, even when Fe leaching is low from the solid matrix, transient dissolved Fe species can dominate early-time kinetics, complicating structure–activity relationships [8]. Furthermore, while oxidative treatments may stabilize Fe species, they risk triggering interlayer collapse, which reduces swelling and impairs mass transfer. Additionally, the formation of adsorbed by-products and organic fouling can block gallery openings and edge sites, leading to a time-dependent deactivation often not visible in short batch experiments. These challenges necessitate a rigorous characterization of Fe species, allowing a distinction between isolated and clustered sites, oxide(s) vs. exchanged Fe ions, and a thorough assessment of coordination changes after thermal treatment. Crucially, the nature of organic substances retained by the clay after reaction must also be evaluated to understand the long-term catalytic stability [4,7,9].

From a larger environmental perspective, the same materials can address depollution of contaminated waters (e.g., from pesticides, pharmaceuticals, dyes and metal ions) and the detoxification of environmental OP/OS. Recent work on iron-exchanged clays for pesticide removal by the heterogeneous Fenton process highlights the feasibility of using natural bentonites as supports and also shows that many studies employ high-temperature calcination (ca. 500 °C) and acidic aqueous conditions, which may not be fully compatible with low-energy, field-deployable decontamination [6,8]. In parallel, recent years have seen an increase in interest in multifunctional solids that combine adsorption with catalytic cleavage or oxidation of CWAs (both real agents or simulants) under mild conditions. Such processes are explored under different conditions, including aqueous solutions, low-water organic media and even decontamination of solid surfaces. Iron-containing clays have already been proposed as active sorbents for CWA-related decontamination; studies on the structure-activity relationship of these solids have focused on various important material parameters, including the accessibility of redox centers, the balance between hydrolysis and oxidation pathways, and the stability of the active phase in the presence of strongly binding phosphonate/sulphonate products [3,23,24].

In this work, we investigated iron-modified natural bentonites for both environmental remediation and protective decontamination. The raw clay was collected from the Taganskoe deposit in Kazakhstan, where it represents a locally abundant resource [25]. Fe(II)- and Fe(III)-exchanged bentonites were prepared via simple aqueous ion exchange, followed by a thermal treatment to enhance the robustness and stability of the Fe species within the smectite framework. The properties of Fe-modified clays were correlated with their decontamination performance toward dimethyl methylphosphonate (DMMP) and 2-chloroethyl ethyl sulfide (2-CEES), an OP and OS simulant, respectively, under mild conditions. A comprehensive physico-chemical characterization was performed before and after decontamination tests to identify the key aspects governing adsorption and degradation, specifically focusing on their potential usage in real decontamination applications [26,27,28].

## 2. Results and Discussion

### 2.1. Preparation of Fe(II)- and Fe(III)-Intercalated Bentonites

The pristine Ben sample from the Taganskoe deposit in Kazakhstan (CEC of 92.6 ± 0.5 meq/100 g) shows a chemical composition typical of natural montmorillonite clay minerals, mainly consisting of SiO_2_ and Al_2_O_3_, with Fe_2_O_3_, MgO and CaO as secondary main components and with a negligible carbon content (Table S1). The raw Ben clay contains 3.07 wt.% of Fe, while ion exchange procedures with 1M solutions of FeSO_4_·7H_2_O or Fe(NO_3_)_3_·9H_2_O introduce additional iron species up to 6.35 wt.% for Fe(II)-Ben and 5.34 wt.% for Fe(III)-Ben (Table 1). Thermal treatment under air at 200 °C of the Fe-exchanged clays led to a slight increase in total iron content, reaching 6.72 wt.% for Fe(II)-Ben calc and 5.59 wt.% for Fe(III)-Ben calc, respectively. This enrichment is likely related to the incipient loss of water by surface condensation during thermal treatments. These loadings remain consistent with values reported in the literature for Fe-exchanged clays used in heterogeneous oxidative catalysis [6,28].

**Figure 1:**
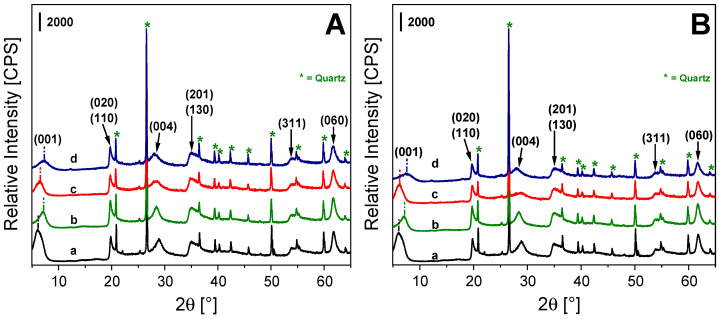
X-ray powder diffraction patterns of Ben (a), Na-Ben (b), Fe-Ben (c) and Fe-Ben calc (d). Frame (**A**) is associated with Fe(III)-exchanged clays, while Frame (**B**) is related to Fe(II)-exchanged clays. Quartz phase is indicated with *.

X-ray powder diffraction patterns of bentonite solids (Figure 1A,B) confirm that montmorillonite is the dominant phase (PDF# 03-0010), with quartz as a secondary component (*, PDF# 46-1045), and indicate that the overall smectite framework is preserved after the exchange procedure with both Fe salts and calcination treatment [29]. The PXRD reflections assigned to the (001), (020)-(110), (004), (201)-(130), (311) and (060) lattice planes in Figure 1A,B, and present in all samples, are typical of a smectite phase with trioctahedral 2:1 T-O-T layered structure (a–d) [30,31,32,33]. The non-negligible presence of quartz in montmorillonite mineral is typical in clays of natural origin. Na-Ben PXRD pattern (Figure 1A,B, curve b) shows a modest shift of (001) reflection (ca. 0.22 nm) after ionic exchange with Na^+^ ions, suggesting a small shrinking of the inter-layer distance of the clay, as observed in the literature for parent clays [34]. No signals related to bulk iron oxide phases are present in PXRD patterns of exchanged clays (Figure 1A,B, curves c, d), thus suggesting a prevalence of exchanged iron species within the interlayer region [35].

**Figure 2:**
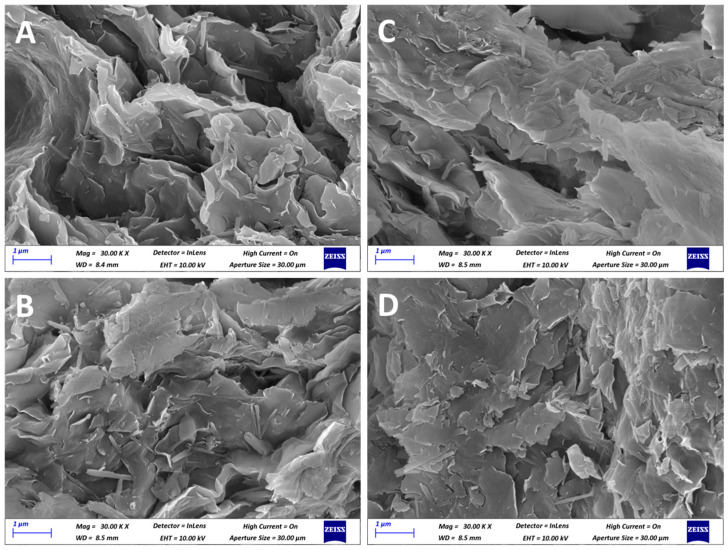
FE-SEM micrographs of Fe(III)-Ben (**A**), Fe(III)-Ben calc (**B**), Fe(II)-Ben (**C**) and Fe(II)-Ben calc (**D**), collected at 30 k magnification.

FE-SEM micrographs of Fe-Ben samples before (Figure 2A,C) and after mild calcination treatment (Figure 2B,D) show the classical platelet-like morphology of bentonite clay [36], which is preserved after the thermal treatment. EDX mapping analysis (Figure S1) shows a uniform Fe dispersion in both samples, with no evident segregation of Fe domains.

Ben and Na-Ben aqueous dispersions show broad particle size distributions centered between 75 and 100 nm, with polydispersity indices (PDI) of ca. 0.35–0.38. The pristine Ben solid shows a bimodal dispersion, which turns into a monodisperse distribution for Na-Ben, after exchange in NaCl solution, suggesting a slight diminution of larger physical aggregates (Figure S2, curves a and b respectively). Moreover, samples display strongly negative ζ-potentials, equal to −45.1 ± 8.03 and −47.7 ± 6.29 mV for Ben and Na-Ben, respectively. These results indicate the formation of stable colloidal dispersions, with a negatively charged surface favorable to the stabilization of positively charged Fe(II) and Fe(III) species in the interlayer space [37,38,39].

**Figure 3:**
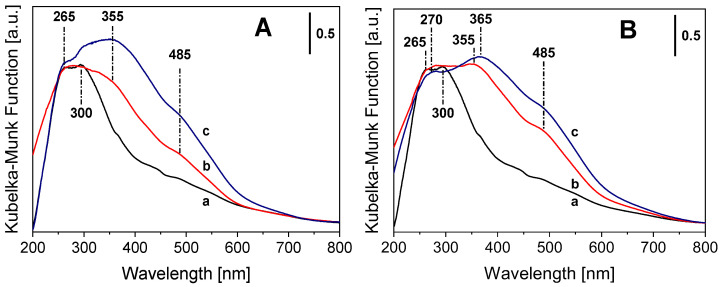
DR UV-Vis spectra of (**A**) Ben (a), Fe(III)-Ben (b), Fe(III)-Ben calc (c), and (**B**) Ben (a), Fe(II)-Ben (b), Fe(II)-Ben calc (c). Measurements have been performed on pure solids.

The coordination state of Fe(III) and Fe(II) in the exchanged bentonite solids was evaluated by DR UV-Vis-NIR spectroscopy. Spectra of Ben and Fe-Ben solids are reported in Figure 3A and Figure 3B for Fe(III) and Fe(II) samples, respectively. The UV-Vis spectrum of the Ben sample (Figure 3A,B, curve a) is mainly characterized by a broad adsorption with maxima at ca. 265 and 300 nm that can be ascribed to ligand-to-metal charge transfer (LMCT) transitions from O^2−^ to isolated Fe(III) ions, as observed in other Fe-doped alumino-silicate systems in octahedral sites [24,40,41,42]. The complex adsorption above 300 nm can be related to the presence of iron oxy/hydroxide clusters with different dimensions [43]. Indeed, bands in the 300–400 nm region correspond to CT transitions in small oligomers, whereas bands extending beyond 400 nm arise from larger nanocrystals and *d*-*d* transitions [24,44]. The ion exchange with iron species (both Fe(III) and Fe(II) precursors) leads to the formation of a complex absorption above 300 nm with evident peaks in the 355–365 nm range and at ca. 485 nm (Figure 3A,B, curve b). The signals between 355 and 365 nm and at ca. 485 nm in Fe-Ben samples (Figure 3A,B, curves b and c) can be attributed to nano- and micro-sized clusters of iron oxide particles, mainly located on the clay surface, respectively [24,40,41,42]. These absorptions have different relative intensities in Fe(III)- and Fe(II)-exchanged bentonites (Figure 3A,B, curve b), and they appear more pronounced after calcination (Figure 3A,B, curve c), suggesting that the oxidative treatment led to a slight increase in small iron oxide content. In the case of Fe(II)-Ben (Figure 3B, b), the simultaneous presence of the intense UV LMCT band at ca. 265–270 nm and the broad band in the 355–485 nm range suggests the presence of mixed-valence Fe(II)/Fe(III) species together with iron oxide clusters [41,42]. Additionally, Fe(II)-Ben shows a weak absorption at ca. 960 nm, which can be assigned to Fe^2+^ crystal-field transitions (Figure S3b) [45]. While this Fe^2+^ signature band remains essentially unchanged after calcination (Figure S3c), the low-energy absorptions become more pronounced, which may indicate further oxidation of Fe(II) to Fe(III).

### 2.2. DMMP Decontamination Tests

The pristine Ben solid and all Fe-exchanged clays were used to remove DMMP from the liquid phase, as a model compound mimicking the chemical behavior of organophosphorus highly toxic pollutants under mild conditions. The tests were performed in ethyl acetate as the reaction medium, since in soil remediation processes, it is common practice to first elute the soil with organic solvents, then concentrate pollutants and finally proceed to decontaminate pre-concentrated contaminants. In addition, preliminary tests on mixed aqueous-organic media were not promising, due to the formation of unmanageable triphasic mixtures.

Raw Ben (Figure S4) shows a remarkable DMMP removal under the tested conditions, up to 70% of the initial OP concentration already after 0.5 h, indicating that the surface and the interlayer spaces of the natural clay already provide a suitable environment for adsorption-assisted transformation reactions. In the reaction mixture, no evident formation of hydrolyzed OP (by)products was detected, thus suggesting that DMMP removal is predominantly due to strong adsorption phenomena. Then, the sodium-containing clay, Na-Ben, was selected to investigate whether a solid with a more basic character, introduced after ion exchange with NaCl, would have been able to degrade DMMP via a hydrolysis pathway catalyzed by basic sites [46,47,48,49]. In addition, the Na^+^-exchange procedure was carried out to homogenize the interlayer chemical composition of the natural bentonite clay, so that all counter-cations were exclusively Na^+^ (in addition to water) in the final Na-Ben solid.

Nevertheless, Na-Ben displays even lower abatement activity (Figure S4), possibly due to an altered solvation/organization of adsorbed DMMP onto the clay after Na^+^ ion exchange. Likewise, taking into account that pristine Ben material contains a non-negligible amount of Fe (3.07 wt.%, cf. Table 1) and that Fenton-type radical activation mechanisms can benefit from the addition of H_2_O_2_, in one test, an aliquot in excess (1:40 molar ratio) of concentrated (ca. 35%) aqueous H_2_O_2_ was added to the reaction mixture. However, no improvement was observed and the maximum removal could hardly attain 40% (Figure S4).

**Figure 4:**
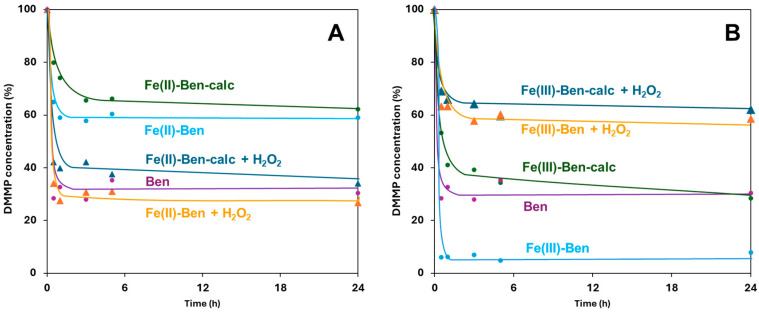
DMMP concentration (mol%) vs. time over: (**A**) Ben, Fe(II)-Ben and Fe(II)-Ben calc, with/without addition of H_2_O_2_; (**B**) Ben, Fe(III)-Ben and Fe(III)-Ben calc, with/without addition of H_2_O_2_. The curves were extrapolated from the GC–FID data. Experimental conditions: 220 ppm DMMP in EtOAc, 80 mg clay, 25 °C, 1 atm; when present, H_2_O_2_ was added at a DMMP:H_2_O_2_ molar ratio of 1:40.

Fe-exchanged Ben solids, on the other hand, show variable performance with differences based on sample type and the presence/absence of H_2_O_2_ (Figure 4A,B). Fe(III)-Ben (Figure 4B) achieves a very high DMMP removal (>93% after 0.5 h), outperforming the result obtained over the pristine Ben material. This behavior is consistent with a hydrolytic DMMP degradation pathway promoted by Fe(III)-based Lewis acidity and bound hydroxyls [24,50,51], along with the adsorption capability of the clay itself. On the contrary, Fe(II)-Ben (Figure 4A) displays lower performances in this regard [52].

The addition of H_2_O_2_ suppresses DMMP removal over Fe(III)-Ben (attaining ca. 40% maximum), suggesting a competitive adsorption of hydrogen peroxide or blockage of reactive iron sites. Conversely, Fe(II)-Ben benefits from the addition of H_2_O_2_, which is congruent with Fenton-type radical activation mechanisms [6,9,21]. Fe-Ben calc samples show reduced activities relative to their non-calcined counterparts, even with the addition of H_2_O_2_. The results obtained with the prepared Fe-exchanged bentonites are consistent with prior reports on Fe-modified clays for pesticide and organic pollutant degradation [6,8].

**Figure 5:**
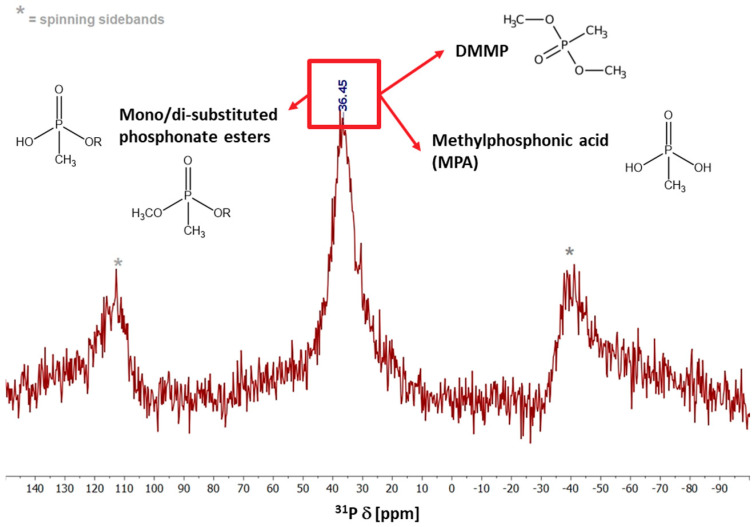
^31^P MAS ssNMR spectrum of Fe(III)-Ben after 5 h of reaction with DMMP. * denotes spinning sidebands.

CHN analysis on Fe(III)-Ben solid after 5 h of reaction with DMMP shows an increased carbon content accumulation (from 0.20 to 1.04 wt.%), which is consistent with the adsorption of DMMP/phosphonate (by)products at the surface as well as in the interlayer spaces of the clay. Indeed, the ^31^P MAS solid state NMR spectrum of Fe(III)-Ben after DMMP contact (at the end of 5 h) (Figure 5) shows a broad signal centered around 36 ppm associated with the possible presence of DMMP and its reaction (by)products, such as mono/di-substituted phosphonate esters and methylphosphonic acid (MPA), adsorbed on the solid [52,53,54,55]. This corroborates the effective degradation of DMMP during tests, as inferred from the decontamination tests’ data. Hence, clay simultaneously acts as a catalyst and adsorbent, which can greatly affect the DMMP abatement activity over time. Indeed, (i) phosphonate products can coordinate to Fe, block active sites and increase hydrophobicity; (ii) labile interlayer Fe ions may be mobilized by complexation, leading to partial Fe loss and the occurrence of homogeneous Fenton-like reactions in heterogeneous conditions [56]. Preventing metal leaching during catalysis while keeping Fe species accessible to redox processes in heterogeneous systems remains an important challenge for the development of efficient materials and decontamination methods [6,57,58]. In addition, PXRD patterns (Figure S5) and EDX mapping analyses (Figure S6), recorded over the spent solids, show a preservation of the layered structure and morphology of Fe(III)-Ben and Fe(III)-Ben calc samples (curves b and d), with a slight shift in the (001) basal plane mainly due to changes in the interlayer hydration state and/or possible leaching of intercalated Fe species.

To get a deeper insight onto the potential loss of Fe species, heterogeneity tests were carried out on the best-performing Fe(III)-Ben solid, by: (i) running the decontamination test for 30 min, (ii) centrifuging the reaction suspension mixture (at 3.5 k rpm), (iii) removing the solid and (iv) adding a fresh aliquot of DMMP (220 ppm) to the supernatant solution. In the absence of the clay, no detectable further removal of DMMP was recorded, thus indicating that abatement activity is largely associated with the solid phase. Likewise, the presence of soluble Fe species in the homogeneous phase was investigated by measuring the total Fe concentration (both Fe(III) and Fe(II)) with colorimetric Fe-bipyridine complexation. These assays showed that a maximum concentration up to 1 ppm of Fe was found in 10 mL reaction mixture, which corresponds to less than 1% of the original Fe content in the fresh Fe(III)-Ben solid. Heterogeneity tests, together with the observed Fe loss, indicate that, while the active phase is predominantly solid-bound to Ben and Fe-Ben clays, we cannot exclude that some labile Fe species may contribute to a transient homogeneous hydrolytic activity during the tests. However, the bulk of the abatement capability likely takes place on the solid clays.

Comparison tests with Fe(III)-exchanged K10 commercial montmorillonite (Fe(III)-K10 mont) and a commercial natural-derived Fe-containing clay, Fulcat (Fulcat clay), show that the Kazakh Fe(III)-Ben solid performs markedly better than commercial analogs under identical conditions (Figure S7). In this case, too, the addition of aq. H_2_O_2_ does not enhance removal capability; rather, it leads to scarcer results. Moreover, it is worth noting the unusual trend of the DMMP concentration profile observed over Fulcat clay, which shows a marked initial drop in concentration after 1 h and, then, a smooth increase in DMMP content in solution. This suggests a limited adsorption capability of this kind of clay with respect to Fe-Ben solids, over which no clear peaks of the phosphorus (by)products were recorded in GC analysis. Such peculiar behavior can be attributed to the different chemical environment of the Fe sites in Fulcat clay. Indeed, as shown by the DR UV-Vis spectrum of this solid (Figure S8a), iron sites are mostly located in an octahedral configuration, and they are, thus, likely located in the octahedral sheets of the lamellae, making them less accessible by DMMP substrate molecules for both adsorption and degradation. So, aiming for a clay suitable for depollution purposes, the combined adsorption and catalytic hydrolysis properties of Fe-Ben clays are optimal. These results suggest that natural bentonites from Kazakh Taganskoe deposits may be a viable low-cost precursor for obtaining pollution remediation solids through a simple ion exchange step with iron salts.

### 2.3. 2-CEES Decontamination Tests

**Figure 6:**
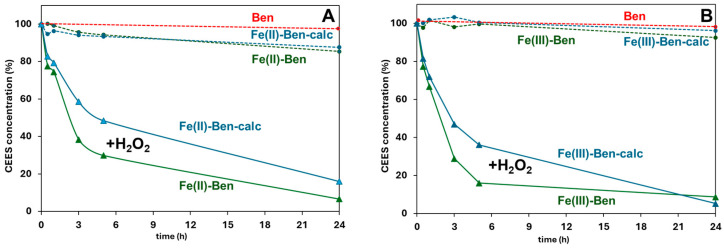
2-CEES concentration (mol%) vs. time over: (**A**) Ben, Fe(II)-Ben and Fe(II)-Ben calc, with/without addition of H_2_O_2_; (**B**) Ben, Fe(III)-Ben and Fe(III)-Ben calc, with/without addition of H_2_O_2_. The curves were extrapolated from the GC–FID data. Experimental conditions: 16.4 μL of 2-CEES in EtOAc, 80 mg clay, 25 °C, 1 atm; when present, H_2_O_2_ was added at a 2-CEES:H_2_O_2_ molar ratio of 1:5.

The entire set of Fe-exchanged Ben clays was applied to the removal of 2-CEES from the liquid phase, as a model compound exhibiting the chemical behavior of the highly toxic blister agent (HD) under mild conditions (Figure 6). In these tests, abatement over clay samples is generally negligible without hydrogen peroxide as an oxidant. The same takes place in the presence of H_2_O_2_, but with no solid. On the contrary, H_2_O_2_ addition (1:5 2-CEES:oxidant molar ratio) enhances performance across all Fe-exchanged clays, with several formulations exceeding 90% conversion at 24 h. This behavior is consistent with the occurrence of an oxidative detoxification pathway of the target compound, as previously observed in the literature for the degradation of OS simulants over clay and polyoxometalate materials under similar experimental conditions, enabling the conversions of the sulfide to sulfoxide and sulfone [59,60]. Thus, Fe-exchanged bentonites can activate the peroxide efficiently under mild conditions (ambient temperature and pressure) to promote a safe degradation of the sulfur mustard simulant 2-CEES.

A comparison test between Fe(III)-Ben and the commercial Fulcat clay was performed on this substrate, too (Figure S9). In terms of activity, the 2-CEES conversion was higher over Fulcat clay in the first hour of reaction, only in the presence of H_2_O_2_. Then, for longer reaction times, the differences tend to overlap, reaching an overall ca. 93–95% conversion at 24 h. In this case too, Fulcat clay is less prone to retain the substrate and the reaction (by)products. Over this solid, clear peaks of the pristine sulfide and the oxidized products (sulfoxide and sulfone, Scheme S1) were recorded in GC analysis [61]. Conversely, over Fe-Ben materials, only negligible amounts of organosulfur (by)products were found in homogeneous solution, hence confirming the good depollution capability, thanks to the synergy between adsorption and catalytic degradation of the undesired hazardous compounds. Moreover, in the specific case of organosulfur oxidation products, the presence, in the final reaction mixture, of 2-chloroethylethylsulfone (Scheme S1) is particularly detrimental, since the sulfone derived from overoxidation of the sulfur mustard blister CWA simulant is almost as toxic as the original sulfide itself.

## 3. Materials and Methods

### 3.1. Chemical Reagents

Natural raw bentonite (Ben) was obtained from the Taganskoe deposit (Kazakhstan). Commercial montmorillonite K10 (Merck, Darmstadt, Germany) and Fe-containing montmorillonite clay (Fulcat 22B; BYK, Wesel, Germany) were used as references in selected control tests. Fe(NO_3_)_3_·9H_2_O (≥99%, Merck, Darmstadt, Germany) and FeSO_4_·7H_2_O (≥99.95%, Merck, Darmstadt, Germany) salts were used as precursors for Fe(III) and Fe(II) exchange processes; NaCl (≥99%, Merck, Darmstadt, Germany) salt was used for Na-exchanged bentonite (Na-Ben). Dimethyl methylphosphonate (DMMP, Merck, Darmstadt, Germany) and 2-chloroethyl ethyl sulfide (2-CEES, TCI, Tokyo, Japan) were used as OP/OS CWA simulants. Ethyl acetate (EtOAc; Merck, Darmstadt, Germany) served as solvent and dodecane as internal standard. Hydrogen peroxide (34.5–36.5 wt.% aq. solution; Merck, Darmstadt, Germany). The pristine natural clays, as received from the manufacturer, were washed with ultrapure Millipore MilliQ^®^ (Merck, Darmstadt, Germany) water, re-ground and dried in an oven at 110 °C for 24 h before use or prior to the intercalation procedure. Further, the solids were homogenized in terms of particle size using standard sieves (mesh 35/45).

### 3.2. Preparation of Exchanged Clays

Na-Ben was prepared by contacting Ben with a saturated NaCl solution for 36 h at rt at a 1 g:100 mL ratio, followed by extensive washing with distilled water and drying [62,63,64]. Fe(II)-Ben and Fe(III)-Ben solids were obtained by a similar ion exchange procedure with 1 M FeSO_4_·7H_2_O and 1 M Fe(NO_3_)_3_·9H_2_O solutions, respectively, followed by washing to remove excess salts and drying. Calcined samples (indicated with “-calc” in sample codes) were obtained by thermal treatment under air at 200 °C for 2 h. In the sample names, Fe(II) and Fe(III) indicate the nature of the Fe precursor used in the exchange process, not the actual oxidation state of Fe in the final solid.

### 3.3. Decontamination Tests

Reactions were performed at 25 °C and 1 atm in EtOAc suspensions (80 mg catalyst, 10 mL solution). For DMMP tests, 220 ppm DMMP was used; when present, H_2_O_2_ was added at a DMMP:H_2_O_2_ molar ratio of 1:40 [3]. For 2-CEES tests, 16.4 μL 2-CEES were added to 10 mL EtOAc; when present, H_2_O_2_ was added at a 2-CEES:H_2_O_2_ ratio of 1:5 [24]. Time-resolved concentration conversion profiles were obtained by GC–FID.

### 3.4. Characterization

Fe loading values were determined by energy dispersive X-ray fluorescence spectroscopy (ED-XRF), using a Rigaku NEX CG II series 3D cartesian geometry ED-XRF spectrometer (Tokyo, Japan) operating with a high-power X-ray tube (50 kV−50 W/65 kV−100 W) and a large-area high-throughput silicon drift detector (SDD), analyzing Na to U elements. The instrument is equipped with a vacuum/helium purge, automatic sample changers and a sample spinner tray.

CHN analyses were carried out on bentonite and Fe(III)-bentonite samples before and after catalysis to assess the organic content, using an EuroVector EA3000 CHN Elemental Analyzer (Milan, Italy). Acetanilide, purchased from EuroVector, was used as a calibration standard (C % = 71.089, H % = 6.711, N % = 10.363).

Powder X-ray diffraction (PXRD) was used for phase identification and to monitor basal *d*_(001)_ spacing changes, using a Bruker D8 Advance powder diffractometer (Karlsruhe, Germany) with Bragg- Brentano geometry, operating with a Cu-Kα_1_ monochromatic radiation (λ = 1.5406 Å) at 40 kV and 40 mA; the instrument is also equipped with a Ni filter. The 2θ range explored was 5–65°, with 2θ steps of 0.02°, 0.1 s/step and automatic synchronization of the anti-scatter knife.

Field-emission scanning electron microscopy (FE-SEM) was used to probe morphology and Fe dispersion, and also to obtain the chemical composition of the raw bentonite. Micrographs were collected with a ZEISS GeminiSEM 360 (Oberkochen, Germany), equipped with a Schottky field effect emitter as an electron source and an energy dispersive X-ray (EDX) elemental analyzer/detector. Prior to analysis, a conductive Pt-coating (ca. 25 nm) was deposited on samples by chemical vapor deposition (CVD) using the EMITECH K575 Sputter-Coater (Crawley, UK).

Dynamic light scattering (DLS) particle distributions and ζ-potential values were measured in aqueous dispersions (1 mg/mL) to evaluate surface charge and colloidal stability. Analyses were performed using a Malvern Zetasizer Nano ZS (Malvern Panalytical, Malvern, UK), operating in the particle size range of 0.6 nm to 6 µm and equipped with a He-Ne laser (λ = 633 nm).

Diffuse reflectance (DR) UV-Vis-NIR spectra of bentonite and Fe-bentonites were collected on pure solids with a PerkinElmer Lambda 900 double-beam spectrophotometer (Waltham, MA, USA) in the range of 200–2500 nm, with a resolution of 1 nm.

Gas chromatography coupled with a flame ionization detector (GC-FID) was used to monitor the OP/OS simulant conversion in the presence of iron-intercalated bentonites. Analyses were performed with an Agilent 6890 GC equipped with a flame-ionization detector (Santa Clara, CA, USA) (column Agilent HP-5, 19091J-413—30 m × 0.32 mm ID × 0.25 μm, split mode, He carrier gas, column head pressure 150 kPa).

The Fe(III)-Ben solid after DMMP decontamination test was analyzed by ^31^P solid-state nuclear magnetic resonance spectroscopy (ssNMR) to identify adsorbed phosphorus-containing reaction by-products. The 1D spectrum was acquired using a Bruker Avance III 500 Spectrometer (Rheinstetten, Germany) equipped with a wide-bore 11.74 T magnet and a 4 mm triple-resonance probe in double-resonance mode. The operating frequencies for ^1^H and ^31^P were 500.13 and 202.46 MHz, respectively. The powder sample was packed in a zirconia rotor, sealed with a Kel-F cap and spun at a magic angle spinning (MAS) rate of 15 kHz at 300 K. A ^31^P high-power decoupling with magic angle spinning (HPDec-MAS) NMR spectrum was acquired with a 90° pulse and the magnitude of the radiofrequency field was 83 kHz with ^1^H decoupling during acquisition. The spectrum was recorded using 8192 transients with a relaxation delay between accumulations of 30 s. Chemical shifts are reported using the δ scale and are externally referenced to ammonium dihydrogen phosphate at 0.8 ppm for ^31^P.

Iron content in the homogeneous phase was measured by colorimetric kit test (Supelco, MQuant 111136, Darmstadt, Germany), where all Fe ions are reduced to Fe(II) species with hydroxylammonium chloride, which then, in a buffered medium, react with 2,2-bipyridine to form a red complex.

Cationic Exchange Capacity (CEC) was determined according to the standardized method based on hexaamminecobalt trichloride solution and Ultraviolet-Visible spectrophotometry (UV-Vis) [65]. In detail, 0.200 g of Na-Ben was exchanged with 10 mL of 0.02 M hexaamminecobalt(III) chloride ([Co(NH_3_)_6_]^3+^) solution (0.2 mmol) at 25 °C, stirring the mixture for 60 h. After separation by centrifugation, the supernatant sample was analyzed by UV-Vis spectroscopy. The experiments were repeated three times for statistical purposes. UV-Vis spectra were recorded at 25 °C in the range 300–600 nm with a resolution of 1 nm. The absorbance of the band at 475 nm (^1^A_1g_ → ^1^T_1g_) [66], relative to a *d*-*d* spin-allowed Laporte-forbidden transition of Co^3+^, was evaluated to quantify the amount of Co^3+^ ions free in solution, thereby determining the amount exchanged in the process and thus the CEC of the bentonite clay. Standard aqueous [Co(NH_3_)_6_]^3+^ solutions, in the concentration range of 0.05–0.005 M, were measured at 25 °C.

## 4. Conclusions

Natural bentonite from the Taganskoe deposit in Kazakhstan can be transformed into efficient, versatile decontamination solids by a straightforward Fe(II)/Fe(III) ion-exchange procedure that, while increasing the total iron content to ca. 5–7 wt.%, maintains the pristine smectite layered structure. A mild thermal treatment under air (200 °C) modifies the iron coordination environment and slightly increases the fraction of surface iron-oxide clusters, but does not generate detectable bulk oxide phases. Decontamination tests in liquid phase with organophosphorus and organosulfur hazardous chemicals simulants, under very mild conditions, showed a marked dependence on iron oxidation state and availability of an oxidant, such as aqueous hydrogen peroxide. Indeed, Fe(III)-exchanged bentonite promotes rapid DMMP removal in the absence of oxidants (up to >93% after 0.5 h). Over these exchanged clays, DMMP abatement is therefore due to a joint effect of hydrolytic degradation and adsorption of the contaminant. When excess H_2_O_2_ is present, competitive adsorption and/or site blocking likely take place, leading to lower depollution capability. On the contrary, Fe(II)-exchanged bentonites were not promising, as they did not show better results than the original raw Ben clay itself. Post-reaction analyses reveal adsorption of phosphonate products, identifying stability as the main development target for translation toward water depollution and field decontamination applications.

For 2-CEES, the presence of H_2_O_2_ as a reactant is essential and multiple Fe-containing formulations achieve >90% removal within 24 h. Also in this case, the clay matrix efficiently retains oxidation products within the solid and prevents the accumulation of potentially hazardous (by)products in the liquid phase, which is a desirable feature for practical depollution. Low iron release (<1 ppm, <1% of the initial Fe content) and post-reaction structural integrity support the predominance of heterogeneous degradation pathways.

Overall, Fe-exchanged natural bentonites emerge as low-cost, robust platforms in which adsorption and catalytic transformation can be balanced and tuned, providing a promising basis for field-relevant remediation and decontamination under mild conditions.

## Figures and Tables

**Table 1:** Clay materials prepared and used in the present work. Iron content of the Fe-exchanged samples (in wt.%), as derived from ED-XRF analysis. (^a^) Fe(II) and Fe(III) indicate the nature of the Fe precursor used in the exchange process, not the actual oxidation state of Fe in the final solid.

Sample ^a^	Ion Exchange Precursor Solution	Fe Content (wt.%)
Ben	-	3.07
Na-Ben	NaCl saturated	(3.07)
Fe(II)-Ben	FeSO_4_·7H_2_O 1M	6.35
Fe(III)-Ben	Fe(NO_3_)_3_·9H_2_O 1M	5.34
Fe(II)-Ben-calc	FeSO_4_·7H_2_O 1M	6.72
Fe(III)-Ben-calc	Fe(NO_3_)_3_·9H_2_O 1M	5.59

## Data Availability

The original contributions presented in this study are included in the article/Supplementary Materials. Further inquiries can be directed to the corresponding author.

## Supplementary Materials

The following supporting information can be downloaded at https://www.mdpi.com/article/10.3390/molecules31101771/s1: Figure S1: FE-SEM-EDX analyses of Fe(III)-Ben (A), Fe(III)-Ben calc (B), Fe(II)-Ben (C) and Fe(II)-Ben calc (D), with respective Fe EDX distribution maps; Figure S2: Histograms of particle hydrodynamic diameter distributions by number (in [%]) of Ben (a) and Na-Ben (b) in water (1 mg/mL), measured by DLS analyses; Figure S3: DR UV-Vis-NIR spectra of Ben (a), Fe(II)-Ben (b) and Fe(II)-Ben calc (c) in the 200–1300 nm region. Measurements have been performed on pure solids; Figure S4: DMMP concentration profiles vs. time over: Ben, Ben + H_2_O_2_ and Na-Ben. The curves were extrapolated from the GC–FID data. Experimental conditions: 220 ppm DMMP in EtOAc, 80 mg catalyst, 25 °C, 1 atm; when present, H_2_O_2_ was added at DMMP:H_2_O_2_ molar ratio of 1:40; Figure S5: X-ray powder diffraction patterns of Fe(III)-Ben (a), Fe(III)-Ben after exposure to DMMP (5 h) (b), Fe(III)-Ben calc (c) and Fe(III)-Ben calc after exposure to DMMP (5 h) (d). Quartz phase is indicated as * in the pattern; Figure S6: FE-SEM-EDX analyses of Fe(III)-Ben (A) and Fe(III)-Ben calc (B) after 5 h of reaction with DMMP, with respective Fe distribution maps; Figure S7: DMMP concentration profiles vs. time over: Fe(III)-Ben, Fe(III)-K10 mont and Fulcat clay, with/without addition of H_2_O_2_. The curves were extrapolated from the GC–FID data. Experimental conditions: 220 ppm DMMP in EtOAc, 80 mg catalyst, 25 °C, 1 atm; when present, H_2_O_2_ was added at DMMP:H_2_O_2_ molar ratio of 1:40; Figure S8: UV-Vis-NIR diffuse reflectance spectra of Fulcat clay (a), Fe(II)-Ben (b) and Fe(III)-Ben (c). Measurements have been performed on pure solids; Figure S9: 2-CEES concentration profiles vs. time over: Fe(III)-Ben, Fe(II)-Ben and Fulcat clay, with/without addition of H_2_O_2_. A reference curve in the absence of solid (H_2_O_2_ only) was added. The curves were extrapolated from the GC–FID data. Experimental conditions: 16.4 μL of 2-CEES in EtOAc, 80 mg clay, 25 °C, 1 atm; when present, H_2_O_2_ was added at 2-CEES:H_2_O_2_ molar ratio of 1:5; Table S1: Major oxide composition and C, H, N values of bentonite (Ben) from Taganskoe (Kazakhstan) deposit (in wt.%), obtained from EDX and CHN elemental analyses, respectively; Scheme S1: Main degradation pathway of 2-CEES in the presence of the commercial Fulcat clay catalyst and H_2_O_2_.

## Abbreviations

The following abbreviations are used in this manuscript:

ED-XRF	Energy dispersive X-ray fluorescence spectroscopy
PXRD	Powder X-ray diffraction
FE-SEM	Field-emission scanning electron microscopy
EDX	Energy dispersive X-ray spectroscopy
DLS	Dynamic light scattering
DR UV-Vis-NIR	Diffuse reflectance ultraviolet-visible-near-infrared spectroscopy
UV-Vis	Ultraviolet-Visible spectroscopy
GC	Gas chromatography
FID	Flame ionization detector
ssNMR	Solid-state nuclear magnetic resonance spectroscopy
MAS	Magic-angle spinning
HPDec	High-power decoupling
OP	Organophosphorus
OS	Organosulfur
DMMP	Dimethyl methylphosphonate
2-CEES	2-chloroethyl ethyl sulfide
MPA	Methylphosphonic acid
CWA	Chemical warfare agent
HD	Distilled mustard (blister agent)
ROS	Reactive Oxygen Species
EtOAc	Ethyl acetate
CEC	Cation-exchange capacity
PILC	Pillared interlayered clay
T-O-T	Tetrahedral–Octahedral–Tetrahedral
PDI	Polydispersity index
LMCT	Ligand-to-metal charge transfer
