# Supplementary material for: Fe-Exchanged Natural Bentonites from Kazakhstan as Multifunctional Solids for Decontamination from Hazardous Chemicals: Structure–Reactivity Relationships Under Mild Conditions
Source: Molecules. 2026 May 21;31(10):1771. doi: 10.3390/molecules31101771 (PMC13210050; doi:10.3390/molecules31101771)
Supplement: Supplementary file 1 [file molecules-31-01771-s001.zip › molecules-4309990-supplementary.pdf]

molecules

MDPI

Article

Fe-exchanged natural bentonites from Kazakhstan as multifunctional solids for decontamination from hazardous chemicals: structure–reactivity relationships under mild conditions

Stefano Econdi [1], Sholpan Nazarkulova[1,2], Stefano Marchesi [3], Chiara Bisio [1,3], Mukhambetkali Burkitbayev [2] and Matteo Guidotti*[1]

[1] CNR–SCITEC, Institute of Chemical Sciences and Technologies "Giulio Natta", Via C. Golgi 19, 20133 Milan, Italy; stefano.econdi@scitec.cnr.it (S.E.); matteo.guidotti@scitec.cnr.it (M.G.)

[2] Al-Farabi Kazakh National University, Almaty, Kazakhstan; sholpan.nazarkulova@kaznu.edu.kz (S. N.); mukhambetkali.Burkitbayev@kaznu.edu.kz (M.B.)

[3] Department of Sciences and Technological Innovation, University of Eastern Piedmont "A. Avogadro", Viale T. Michel 11, 15121 Alessandria, Italy; stefano.marchesi@uniupo.it (S.M.); chiara.bisio@uniupo.it (C.B.)

* Correspondence: matteo.guidotti@scitec.cnr.it; Tel.: +39 02 50314428

Abstract

Iron-exchanged bentonites derived from a natural montmorillonite-rich clay (Taganskoe deposit, Kazakhstan) were prepared through a simple aqueous ion-exchange route using Fe(II) or Fe(III) inorganic salt precursors, yielding final Fe contents of ca. 5–7 wt.%, while preserving the smectite layered framework. A mild thermal treatment under air was applied to tune iron coordination without triggering major structural collapse. The resulting materials were characterized by ED-XRF, XRD, FE-SEM/EDX, DLS/ζ-potential and DR UV–Vis–NIR spectroscopy, revealing predominantly exchanged Fe species with a limited fraction of surface iron-oxide clusters, whose contribution increases after activation. Structure–reactivity relationships were probed under mild conditions in liquid-phase ethyl acetate using dimethyl methylphosphonate (DMMP) and 2-chloroethyl ethyl sulfide (2-CEES) as organophosphorus and organosulfur hazardous chemicals and chemical warfare agent simulants, respectively. Fe(III)-bentonite enabled very fast DMMP removal (ca. 93% within 0.5 h) with a remarkable improved performance with respect to Fe(II)-bentonite and the pristine mineral clay. For 2-CEES, the presence of H_2O_2 markedly enhanced oxidation on Fe-containing clays, reaching quantitative abatement within 24 h (up to >90%), with strong retention of oxidized sulfur products by the clay matrix. These results highlight Fe-exchanged natural bentonites as robust, cheap and multifunctional adsorption/catalytic solids for decontamination and water-treatment applications.

Keywords: clays; montmorillonite; ion exchange; iron; heterogeneous oxidation; decontamination; organophosphorus chemicals; organosulfur compounds

Academic Editor: Susana Valencia

Received: 24 April 2026
Revised: 15 May 2026
Accepted: 19 May 2026
Published: 21 May 2026

Copyright: © 2026 by the authors. Submitted for possible open access publication under the terms and conditions of the Creative Commons Attribution (CC BY) license.

1. Figures

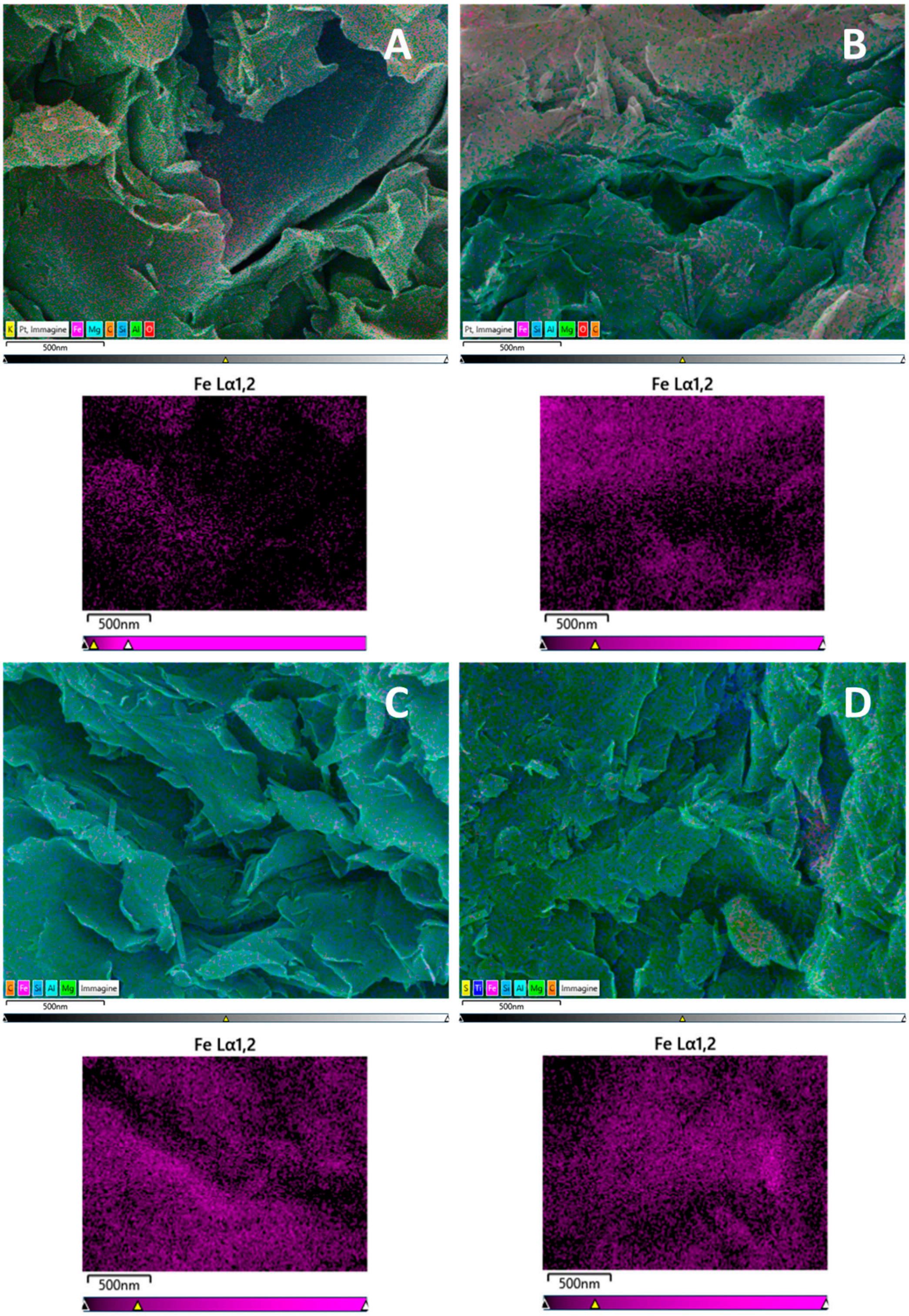

Figure S1. FE-SEM-EDX analyses of Fe(III)-Ben (A), Fe(III)-Ben calc (B), Fe(II)-Ben (C) and Fe(II)-Ben calc (D), with respective Fe EDX distribution maps.

https://doi.org/10.3390/molecules31101771

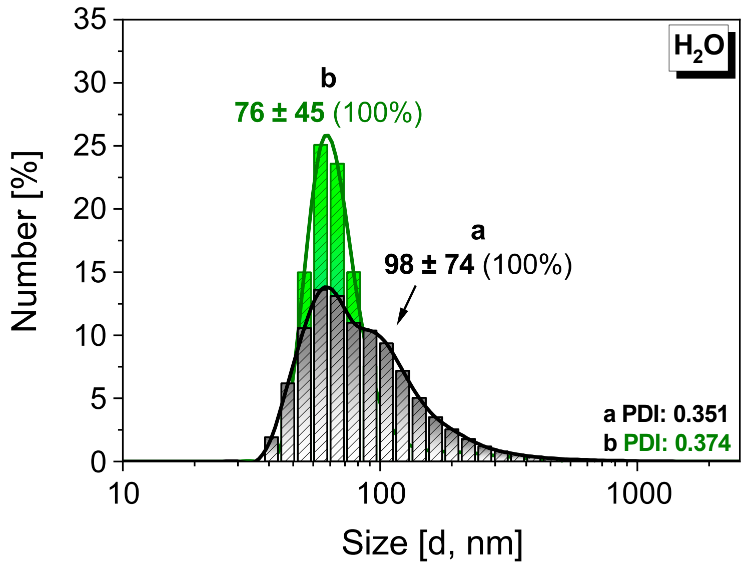

Figure S2. Histograms of particle hydrodynamic diameter distributions by number (in [%]) of Ben (a) and Na-Ben (b) in water (1 mg/mL), measured by DLS analyses.

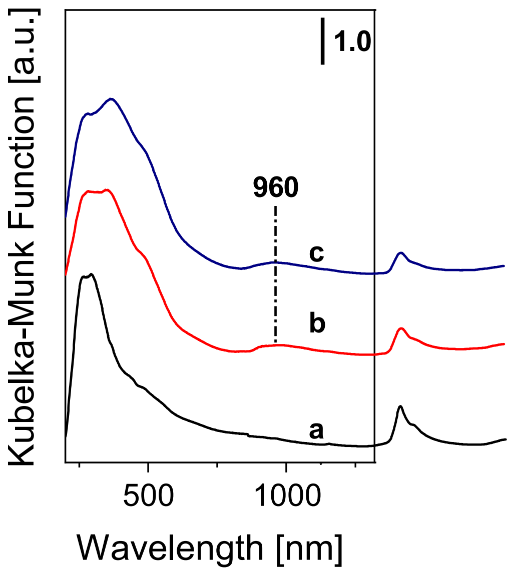

Figure S3. DR UV-Vis-NIR spectra of Ben (a), Fe(II)-Ben (b) and Fe(II)-Ben calc (c) in the 200-1300 nm region. Measurements have been performed on pure solids.

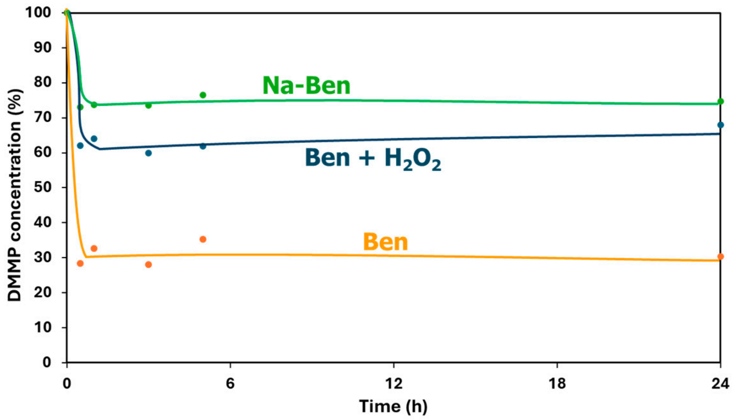

Figure S4. DMMP concentration (mol%) profiles *vs.* time over: Ben, Ben + H_2O_2 and Na-Ben. The curves were extrapolated from the GC–FID data. Experimental conditions: 220 ppm DMMP in EtOAc, 80 mg catalyst, 25 °C, 1 atm; when present, H_2O_2 was added at DMMP:H_2O_2 molar ratio of 1:40.

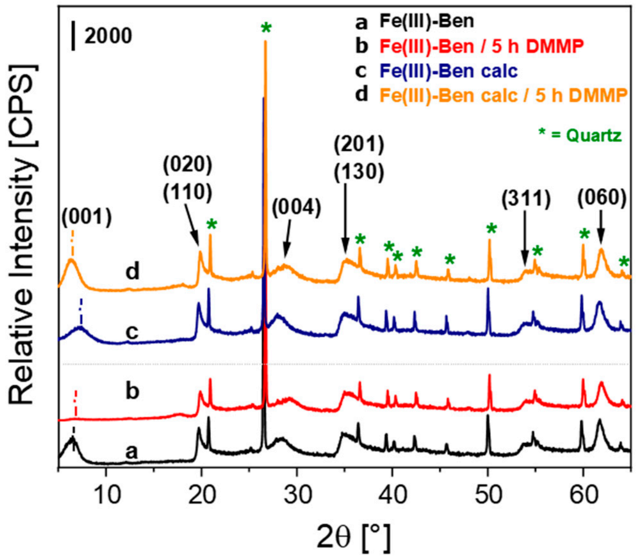

Figure S5. X-ray powder diffraction patterns of Fe(III)-Ben (a), Fe(III)-Ben after exposure to DMMP (5 h) (b), Fe(III)-Ben calc (c) and Fe(III)-Ben calc after exposure to DMMP (5 h) (d). Quartz phase is indicated as * in the pattern.

https://doi.org/10.3390/molecules31101771

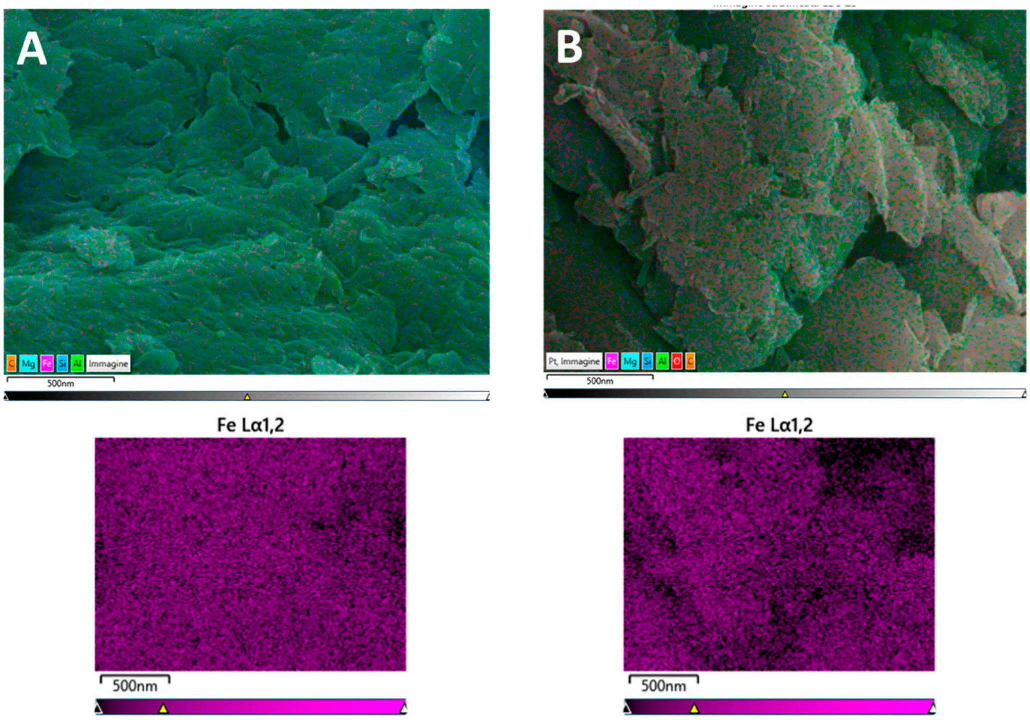

Figure S6. FE-SEM-EDX analyses of Fe(III)-Ben (A) and Fe(III)-Ben calc (B) after 5 h of reaction with DMMP, with respective Fe distribution maps.

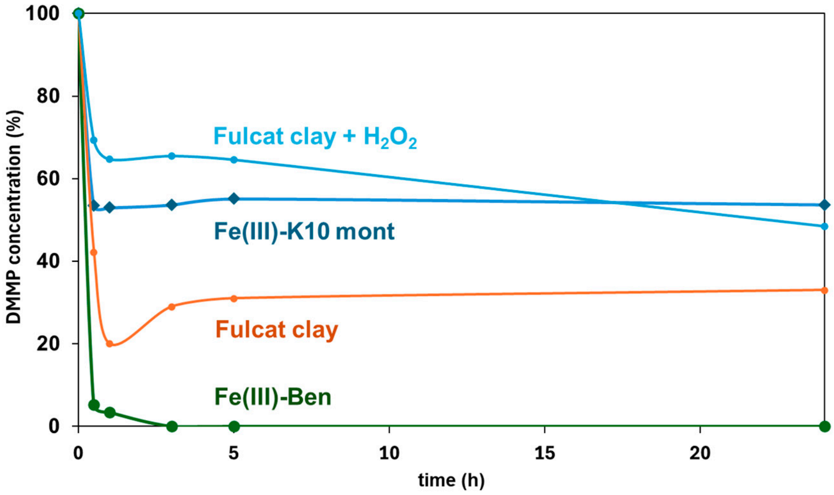

Figure S7. DMMP concentration (mol%) profiles *vs.* time over: Fe(III)-Ben, Fe(III)-K10 mont and Fulcat clay, with/without addition of H_2O_2. The curves were extrapolated from the GC–FID data. Experimental conditions: 220 ppm DMMP in EtOAc, 80 mg catalyst, 25 °C, 1 atm; when present, H_2O_2 was added at DMMP:H_2O_2 molar ratio of 1:40.

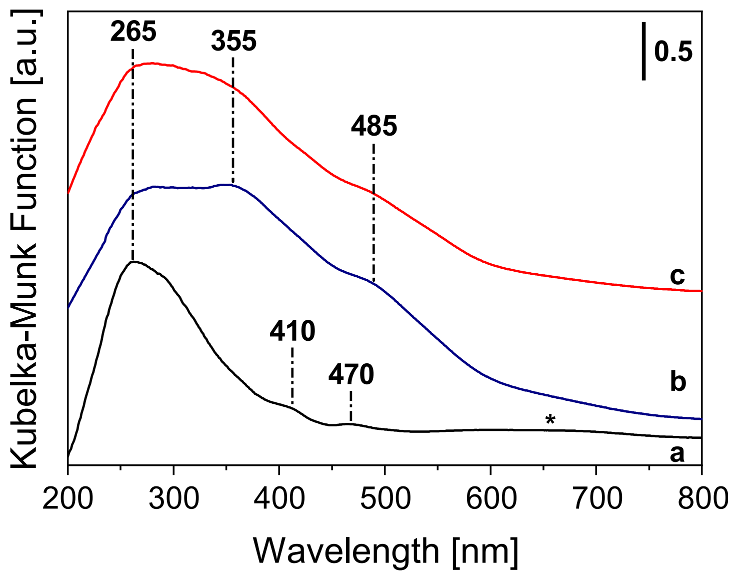

Figure S8. UV-Vis-NIR diffuse reflectance spectra of Fulcat clay (a), Fe(II)-Ben (b) and Fe(III)-Ben (c). Measurements have been performed on pure solids.

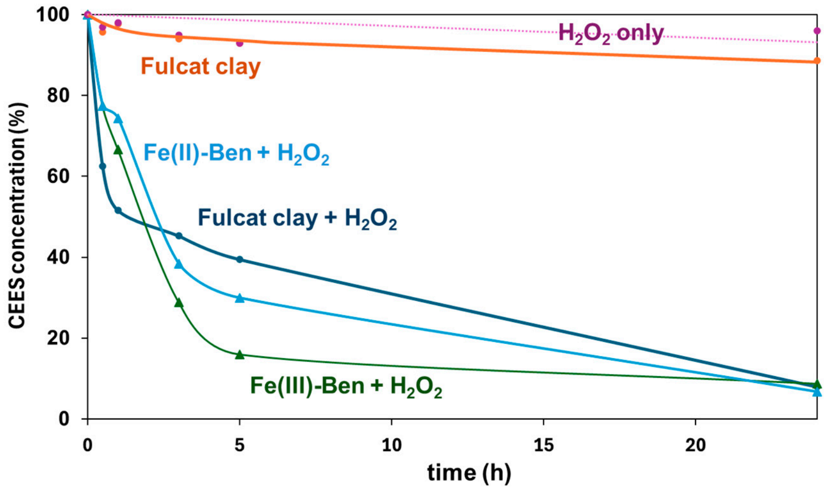

Figure S9. 2-CEES concentration (mol%) profiles *vs*. time over: Fe(III)-Ben, Fe(II)-Ben and Fulcat clay, with/without addition of H_2O_2. A reference curve in the absence of solid (H_2O_2 only) was added. The curves were extrapolated from the GC–FID data. Experimental conditions: 16.4 µL of 2-CEES in EtOAc, 80 mg clay, 25 °C, 1 atm; when present, H_2O_2 was added at 2-CEES:H_2O_2 molar ratio of 1:5.

2. Tables

Table S1. Major oxide composition and C,H,N values of bentonite (Ben) from Taganskoe (Kazakhstan) deposit (in wt.%), obtained from EDX and CHN elemental analyses, respectively.

[wt.%]	SiO_2	Al_2O_3	Fe_2O_3	TiO_2	CaO	MgO	SO_3	K_2O	Na_2O	LOI [1]	C	H	N
Ben	55.50	19.40	4.40	0.30	1.98	2.18	0.20	0.50	0.14	11.30	0.11	1.73	0.00

[1] LOI: loss on ignition.

3. Schemes

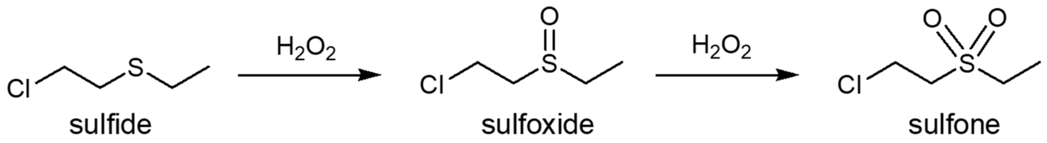

Scheme S1. Main degradation pathway of 2-CEES in the presence of the commercial Fulcat clay catalyst and H_2O_2.

Disclaimer/Publisher's Note: The statements, opinions and data contained in all publications are solely those of the individual author(s) and contributor(s) and not of MDPI and/or the editor(s). MDPI and/or the editor(s) disclaim responsibility for any injury to people or property resulting from any ideas, methods, instructions or products referred to in the content.

https://doi.org/10.3390/molecules31101771
